# Health care providers’ perceived barriers to and need for the implementation of a national integrated health care standard on childhood obesity in the Netherlands – a mixed methods approach

**DOI:** 10.1186/s12913-016-1324-7

**Published:** 2016-03-08

**Authors:** Annemarie A. H. Schalkwijk, Giel Nijpels, Sandra D. M. Bot, Petra J. M. Elders

**Affiliations:** Department of General Practice and Elderly Care and the EMGO Institute for Health and Care Research, VU University Medical Center, Amsterdam, The Netherlands

**Keywords:** Obesity, Child, Primary health care/standards, Qualitative research, Obesity/prevention & control, Integrated health care

## Abstract

**Background:**

In 2010, a national integrated health care standard for (childhood) obesity was published and disseminated in the Netherlands. The aim of this study is to gain insight into the needs of health care providers and the barriers they face in terms of implementing this integrated health care standard.

**Methods:**

A mixed-methods approach was applied using focus groups, semi-structured, face-to-face interviews and an e-mail-based internet survey. The study’s participants included: general practitioners (GPs) (focus groups); health care providers in different professions (face-to-face interviews) and health care providers, including GPs; youth health care workers; pediatricians; dieticians; psychologists and physiotherapists (survey). First, the transcripts from the focus groups were analyzed thematically. The themes identified in this process were then used to analyze the interviews. The results of the analysis of the qualitative data were used to construct the statements used in the e-mail-based internet survey. Responses to items were measured on a 5-point Likert scale and were categorized into three outcomes: ‘agree’ or ‘important’ (response categories 1 and 2), ‘disagree’ or ‘not important’.

**Results:**

Twenty-seven of the GPs that were invited (51 %) participated in four focus groups. Seven of the nine health care professionals that were invited (78 %) participated in the interviews and 222 questionnaires (17 %) were returned and included in the analysis. The following key barriers were identified with regard to the implementation of the integrated health care standard: reluctance to raise the subject; perceived lack of motivation and knowledge on the part of the parents; previous negative experiences with lifestyle programs; financial constraints and the lack of a structured multidisciplinary approach. The main needs identified were: increased knowledge and awareness on the part of both health care providers and parents/children; a social map of effective intervention; structural funding; task rearrangements; a central care coordinator and structural information feedback from the health care providers involved.

**Conclusions:**

The integrated health care standard stipulate that the care of overweight or obese children be provided using an integrated approach. The barriers and needs identified in this study can be used to define strategies to improve the implementation of the integrated health care standard pertaining to overweight and obese children in the Netherlands.

**Electronic supplementary material:**

The online version of this article (doi:10.1186/s12913-016-1324-7) contains supplementary material, which is available to authorized users.

## Background

Childhood obesity is becoming more prevalent around the world and represents an increasingly salient pediatric health concern [1]. The Netherlands has seen a two to three-fold increase in overweight and a four to six-fold increase in obesity since 1980 [2]. In 2009, the prevalence of overweight and obesity amongst children aged 2 to 21 years was 12.8 % and 1.8 % for boys and 14.8 % and 2.2 % for girls, respectively. Overweight and obesity at a young age have important short-and long-term health and social consequences [3]. Obese children have an increased risk of multiple medical co-morbidities [4–7] as well as psychosocial problems [8–10]. Furthermore, childhood obesity has been shown to have a high likelihood of persisting into adolescence and adulthood [1, 11, 12].

Current care for child obesity is constrained by a number of factors. Firstly, care is delivered by a variety of health care professionals and is fragmented, as coordination between health care providers is insufficient [13–16]. Secondly, obese children and their parents experience uncertainty in the care process due to a lack of control and continuity of care [15]. Finally, the health care risks specific to obese children remain unidentified and are insufficiently monitored [15, 17–19].

European guidelines acknowledge the need for a multi-disciplinary approach to the primary and secondary prevention of chronic diseases [20–22]. The internationally recommended treatment of childhood obesity is a family behavioral lifestyle intervention including dietary and physical activity advice and a family-targeted approach in children under 12 years of age [22–24]. While these clinical guidelines do describe the recommended care in some detail, including how, when and by whom care should be provided, they do not specify how this multidisciplinary care should be organized. In its effort to systematically organize the services provided to, and treatment of, children who are overweight or obese on an aggregate level, the Netherlands can be regarded as unique in its use of an integrated health care standard [13]. This integrated health care standard highlights the importance of a central care coordinator whose role it is to oversee the multidisciplinary care process consisting of five key components: 1) identification; 2) diagnosis and risk stratification; 3) individual health care plan and treatment; 4) continuity of care; and 5) multidisciplinary approach [13]. The Cole criteria for childhood obesity were used in the development and dissemination of the integrated health care standard in 2010 [25].

In many European countries, the GP plays an important role in identifying obesity in children and in subsequent interventions [13, 14, 16, 20]. According to the integrated health care standard principles, the majority of overweight and obese children can be managed by GPs, provided that a multidisciplinary team supporting lifestyle changes in children is also available. For only a few obese children with extreme weight-related health risks is referral to specialized health care required [13].

While the integrated health care standard principles specify an ideal of multidisciplinary care of obese children, their feasibility in current practice has never been investigated. It is well established that dissemination alone is unlikely to result in effective implementation in practice; therefore, more active strategies are recommended [16, 26].

Known barriers to implementing change in current practice exist at the level of the individual care provider (e.g. competence, attitude, motivation for change), social setting (e.g. patients, care providers) and system (e.g. organizational structure, financial reimbursement) [26]. Due to the complexity of multidisciplinary care, barriers and needs must first be identified in order to formulate strategies for effective implementation [16, 26]. Understanding the discrepancies between desired and current care is a starting point for initiating change and could generate more support for the implementation of the integrated health care standard on the part of health care providers [26]. Therefore, the aim of this study is to acquire more insight into the barriers to and needs for the implementation of the integrated health care standard as perceived by GPs and other health care providers who manage or treat obesity in children.

## Methods

We have used the consolidated criteria for reporting qualitative research (COREQ) to describe our methods [27].

### Design

We used a mixed-methods technique, combining quantitative and qualitative research [28]. There is broad consensus that mixing different types of methods has the capacity to strengthen a study’s results and conclusions [29]. The barriers to and needs for the implementation of the integrated health care standard are so complex that the use of different kinds of methods was warranted in our efforts to fully account for this complexity. The qualitative study consisted of focus groups with GPs and face-to-face, semi-structured interviews with different health care providers in an effort to identify the barriers to and the necessary requirements for the implementation of the integrated health care standard. A conscious decision was made to keep the nature of the focus groups and face-to-face, semi-structured interviews open in order to allow GPs and health care providers to provide detailed accounts and voluntarily raise issues that were of importance to them. Subsequently, we conducted a large-scale, e-mail-based internet survey amongst health care providers involved in youth health in order to corroborate and complement the qualitative results and increase generalizability. All the health care providers involved were active in the region around Amsterdam.

### Theoretical framework

This study was carried out using Grol and Wensing’s implementation of change model [26]. It focused on the first steps to implementing the integrated health care standard in the Amsterdam area, focusing on the analysis of the context in which changes must occur. It explored barriers and needs grouped according to the five key components of the integrated health care standard: 1) identification; 2) diagnosis and risk stratification; 3) individual health care plan and treatment; 4) continuity of care and 5) a multidisciplinary approach. These components were then divided further into three levels: individual health care providers, social setting and system [26].

### Participants and procedures

Amsterdam, an urban area, was chosen as the research setting, as health care professionals here have experience in the care of obese children due to the implementation of governmental programs and the increasing prevalence of childhood obesity.

### Focus groups and interviews

GPs from the Academic Network of General Practitioners of the VU University Medical Centre in Amsterdam (ANH-VUMC) were invited to participate in the focus groups. The GPs were also asked to identify health care providers to whom they refer obese children. These individuals were then approached by e-mail or telephone and were invited to participate in face-to-face, semi-structured interviews. These health care providers included a Youth Health Care (YHC) nurse, a YHC doctor, a pediatrician, a dietician, a psychologist, a physiotherapist, a social worker, a remedial educationalist and a GP. We choose to approach a variety of health care workers involved in youth care to reflect the integrated health care standard criteria pertaining to multidisciplinary care.

### Internet survey

An e-mail-based internet survey was conducted amongst GPs, dieticians, psychologists, physiotherapists, pediatricians, YHC providers and remedial educationalists. Respondents were approached by email via their different organizations to increase accessibility. These organizations included the principle primary care organization, pediatric departmental secretariats, the management team of the municipal youth health care organization (GG&GD) and various paramedic al services organizations (for dieticians, psychologists, physiotherapists and remedial educationalists).

GPs associated with the ANH-VUMC were only asked to respond to the 21 items in the “importance of need” part of the questionnaire to corroborate their contributions during the focus groups.

Ethical approval for the study was obtained from the VU University Medical Centre Research Ethics Committee. The informed consent procedure was waived by the Ethical Committee. Data collection took place between May 2011 and February 2012.

### Data collection

#### Focus group

The four GP focus groups took place simultaneously in adjoining rooms during the half-yearly meeting of the ANH-VUMC. Each focus group was led by a GP and included at least two observers (vocational GPs) who took notes. We had GPs lead these focus groups to engender mutual confidence between the leaders and participants. The GPs that led the groups were members of the ANH-VUMC research team. Each focus group lasted approximately 60 min, was audio recorded and transcribed verbatim. Each of the groups discussed barriers and needs in the context of current care and the implementation of the integrated health care standard for obese children. Six main questions were developed (Additional file 1) based on the literature on integrated care and the implementation of integrated health care standard.

#### Interview

Each face-to-face, semi-structured interview started with a general introduction and the opening question: “What do you think your role is in the care of obese children?”. First, current care was discussed, followed by an explanation of the integrated health care standard and a discussion of barriers and needs (Additional file 2). At the end of the interview, the researcher ensured that all items had been covered [30]. The interviews were conducted by two trained medical students. Each interview lasted between 60 and 90 min and was audio recorded and transcribed verbatim.

#### E-mail-based internet survey

Statements included in the internet survey were constructed based on the themes that were identified during the analysis of data from both the focus groups and the semi-structured interviews. All statements were measured on a five-point Likert scale ranging from 1 (totally agree) to 5 (totally disagree). Additionally, the survey participants were asked about “importance of need” in the context of 21 themes related to the provision of optimal care according to the integrated health care standard. These themes were constructed after the analysis of the focus group and semi-structured interviews. All statements were measured on a five-point Likert scale ranging from 1 (absolutely not important) to 5 (absolutely important) or on a two-point scale (not important/important). Furthermore, demographic information was gathered for each participant and open questions were included to acquire additional information on experiences and barriers and need related to working according to the integrated health care standard.

### Data analysis

#### Focus group

The transcripts were coded thematically according to the five components of the integrated health care standard and themes that emerged in the discussions. The transcripts of the four focus groups were coded separately by two trained medical students. They then discussed any discrepancies and reached a consensus on the coding. Using a matrix, the codes were then grouped according to the five key components of the integrated health care standard and were divided further into three levels in accordance with the theoretical framework. From these groupings, themes were extracted that represented the main messages conveyed by the focus group data. Themes were identified across the data with regard to the research question [31]. After the analysis had been completed, one researcher (AS) read all of the focus group data and confirmed the themes that had been extracted from the data [30]. The respondents did not provide feedback on the findings.

#### Interview

The themes identified in the focus groups were used to analyze the interviews. The transcripts were coded thematically according to the components of the integrated health care standard. The transcripts of the interviews were coded separately by two trained medical students. Each interview was transcribed and analyzed immediately after the interview to confirm the validity of the theme list. In the subsequent interviews, these themes were developed further with respect to the research question. The themes were ordered using a matrix and were categorized according to the five key components of the integrated health care standard and divided further into three levels in accordance with the theoretical framework. The respondents did not provide feedback on the findings.

#### E-mail-based internet survey

Incomplete questionnaires, surveys completed by underage respondents (e.g. under 21) and those missing information on the respondent’s position were excluded from analysis. Responses to items measured with the 5-point Likert scale were categorized into three outcomes: ‘agree’ or ‘important’ (response categories 1 and 2); ‘disagree’ or ‘not important’ (response categories 4 and 5) and ‘neutral’ (category 3). SPSS version 20.0 for Windows (International Business Machines (IBM) Corp., SPSS Statistics, Armonk, New York, USA) was used to obtain descriptive statistics based on the internet survey.

### Quotes

Quotes were selected on the basis of critical discussion on the part of the research team (AS, PE, GN).

## Results

### Participants

**Fig. 1:**
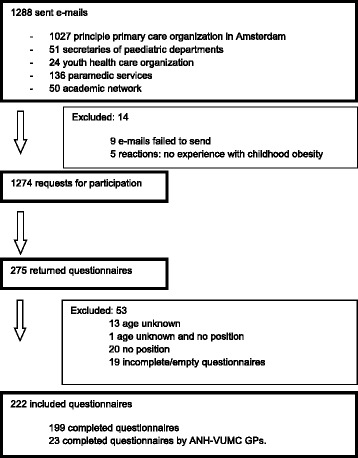
Flow chart of internet survey participants. ANH-VUMC GPs were only asked to respond to the items pertaining to “importance of needs”

A total of 27 GPs (51 %) participated in four focus groups and seven of the nine (78 %) health care professionals (one GP, one YHC nurse, one YHC doctor, one pediatrician, one dietician, one psychologist and one physiotherapist) participated in the interviews. Of the 1288 questionnaires sent, 222 (17 %) were returned and included in the analysis (80 GPs (of which 23 were ANH-VUMC GPs)), 11 YHC workers, 14 pediatricians, 23 dieticians, 13 psychologists, 69 physiotherapists and 12 others (e.g. obesity coordinator). See Fig. 1 for the participant flow chart. The mean age of the professionals in the survey was 45 (23–68 years) and the majority was female (72 %).

Table 1 shows the thematic analysis of the barriers and needs identified by the health care providers’ during the focus groups and the individual interviews. These are grouped according to the five key components of the integrated health care standard. An overview of the survey results is presented in Additional file 3.

**Table 1:** Barriers and needs in the thematic analysis. Indicated by GPs, YHC workers, pediatricians, dieticians, psychologists and physiotherapists during the focus groups and individual interviews

	Barriers^a^	Needs
Component 1: identification		
Individual care provider	Afraid of harming relationship with parents as a result of discussing a child’s weight problem. Afraid of the reaction of obese child while discussing the weight problem.	Increase knowledge and awareness of health care providers in identifying obese children.
System	When society does not change (pro-healthy lifestyle), it feels like a waste of time to identify and treat obese children.	
	No regular screening program for children between 5–10 years of age; therefore children don’t show up for consultation.	Annual screening.
	Reluctance to provide evidence-based care due to insufficient financial compensation.	Financial reimbursement for health care providers by health insurance companies.
Social setting	Not able to discuss weight problem because parents lack knowledge, expertise and obesity awareness.	Education and information leaflets for parents and children.
	Impression that parents lack motivation to attend follow-up appointments, lack disease awareness, lack motivation to change lifestyle and are unaware of the consequences of overweight/obesity.	Increase knowledge and awareness of parents and children.
	Prevalence of multiple-problems families and low socio-economic status of families with obese children.	More time to help these families, which means funding for extra human resources.
Component 2: diagnosis and risk stratification		
Individual care provider	Unfamiliar with weight-related health risk (GGR) and risk stratification.	Clear cut-off points and tools with which to perform a risk stratification (GGR).
System		
Social setting		
Component 3: individual care plan and treatment		
Individual care provider	Time consuming to create an individual care plan.	Social map with an overview of effective lifestyle interventions.
	Negative experience with previous lifestyle intervention.	
System	Difficult to keep all health care providers informed of (temporary) lifestyle interventions.	Financial compensation for lifestyle interventions.
Social setting	Parents and obese children do not enter the lifestyle interventions due to financial constraints.	Financial compensation for sports/fitness facilities.
Component 4: continuity of care		
Individual care provider	Lack of time to monitor and give sufficient attention to parents and obese children.	Financial compensation for continuity of care.
System	Lack of long-term lifestyle interventions. Lack of structural funding for long-term lifestyle interventions.	Reimbursement by insurance company for long-term lifestyle interventions.
Social setting	High drop out rate of children in “expensive (long) term pediatric care”.	
	No insight into the number of children being referred. Changing lifestyle behavior is difficult for parents and obese children.	
Component 5: multidisciplinary approach		
Individual care provider		
System	Lack of collaboration with health care providers involved.	Recruitment of a central care coordinator could enable the provision of multidisciplinary care.
	No clear task (re-)arrangements	Collaboration agreements and task rearrangements with health care providers involved in an region.
		Structural funding needed to provide multidisciplinary care.
Social setting	No collaboration with health care providers involved due to lack of feedback information from health care providers.	Feedback information from health care providers provided.

*GGR* Weight-related health risk

^a^The barriers and needs are grouped into the five key components of the integrated health care standard and divided into three levels, individual care provider (e.g. competence, attitude, motivation for change), system (e.g. organization (structure), financial reimbursement), social setting (e.g. parents/obese children, care providers)

### Interpretation of role in the care of obese children

#### Focus group/interview

All health care providers recognized the importance and necessity of treating obese children. The majority of the GPs indicated that they currently have no role in the care of obese children. Only a few had actually treated obese children. The YHC doctor considered her function to be limited to measuring weight and providing referrals if needed. The pediatrician, the physiotherapist and the dietician claimed only to treat obese children after referral by another health care provider. The pediatrician indicated only having treating obese children with co-morbidities.

#### Survey

Forty percent of the GPs surveyed responded that they saw it as their role to treat children with obesity, whereas 49 % had no clear opinion and 30 % saw it as their role to treat obese children and indirectly their parents as well. Of all health care providers, 58 % were experienced in the care of obese children; for GPs, this number was less than 40 %.

### Barriers and needs

#### Component 1: identification

##### Focus group

GPs indicated four main barriers to identifying obese children.

1. They are reluctant to raise the subject, fearing that it might harm their relationship with the parents or negatively influence the happiness of the child.
2. When they raise the subject and invite parents for follow up appointments, parents often do not show up.
3. They experience a lack of knowledge, expertise and obesity awareness.
4. They believe that parents are unaware of the consequences of obesity and, in general, are not motivated to take action.

The GPs indicated that these barriers could be overcome with education leaflets directed at children/parents in addition to an increase in knowledge, expertise and obesity awareness on the part of GPs.

GP: “I’ve seen children just cringe when discussing their weight problem. That has made me reluctant to discuss the problem.”

##### Interview

All health care providers supported the GPs’ opinion that parents lack knowledge of the consequences of overweight and obesity and lack motivation to take any action. There was consensus about the prevalence of obese children in multiple-problem families and inexperience on the part of GPs in dealing with less-educated families as being major barriers to discussing weight problems. The YHC worker identified the inability to identify obese children in certain age groups as a barrier, since children only have routine check-ups between the ages of 0 and 4 and at the ages of 5, 10 and 13. The dietician also identified the absence of structural screening of children aged between 5 to 10 years as a barrier.

In contrast with the GPs, the pediatrician and YHC indicated that they had no problem raising the subject with children and parents.

GP: “There is a lack of awareness and it’s not enough of a priority in some families. I noticed it more often in troubled families. I also often find it very difficult to help these families and, honestly, I admit I don’t feel equipped to support these children. I find some families to be just too complex.”

Pediatrician: “I have no problem addressing a weight problem. I know what the health consequences may be and often parents also know. The problem is of course that we both know (the parents and I) that it is a persistent problem.”

##### Survey

Less than half of the health care providers surveyed (45 %) were aware of the existence of the integrated health care standard and 35 % were aware of their content, which was still more than was indicated in the focus groups and individual interviews. Most GPs do not have a structured plan to help children with obesity (61 %), nor do they have enough time to do so (60 %). Some GPs (13 %) find it difficult to address obesity and of these GPs, 75 % indicate that parents do not request help with their child’s weight problem; 13 % do not want to harm their relationship with the child/parents and 63 % agree that raising the subject is too time consuming.

When asked how these barriers could be overcome, 75 % of GPs and 70 % of all health care providers agreed that information leaflets for parents and children are needed and 63 % of GPs and 69 % of all health care providers emphasized the importance of an annual screening of weight for children aged 4 to 12 years.

#### Component 2: diagnosis and risk stratification

##### Focus group

GPs indicated they were unfamiliar with the weight-related health risk (GGR) described in the integrated health care standard and how to assess this. Although they were unfamiliar with the GGR, they indicated that if the assessment of weight-related health risk became part of routine care, the GP should have a role in the assessment.

GP: “Yes, the identification of the health risk might be an appropriate task for the GP. Thereafter the dietician, physiotherapist or pedagogue should play a more important role.”

##### Interview

Of all the health care providers who diagnose and perform a risk stratification, only the YHC-doctor indicated using an internet tool to do so. Some indicated the need for a tool to identify psychological problems.

Dietician: “In the hospital, they discuss psychological issues and often talk about low self-esteem in children. We can’t observe this very well, since we ask about it only very superficially. We have no surveys, no scoring lists for things like this like they do in the hospital.”

##### Survey

Most GPs (80 %) and 58 % of the health care providers indicated that they had sufficient tools to diagnose obesity. Sixty-six percent of GPs and 78 % of the health care providers found it important to have additional diagnostic tools at their disposal to identify psychological problems.

#### Component 3: individual care plan and treatment

##### Focus group

GPs indicated seeing the creation of an individual health care plan as a barrier due to being time consuming. Regarding treatment, they were demotivated due to previous negative experience with referrals to behavioral, exercise and nutrition programs. In addition, they had negative experiences with children/parents who did not want to be referred to these programs due to financial constraints. A further barrier was a lack of structural funding for programs that might be effective for these children.

##### Interview

The negative experience of GPs highlighted in the focus groups was shared by other health care providers. Identified needs included a social map with an overview of effective lifestyle interventions and adequate financial compensation for such interventions.

Physiotherapist: “It’s about behavioral change. They need to change their behavior and that’s very hard to do. It’s more than just referring children to the physiotherapist for an exercise program.”

Psychologist: “My previous experience is that someone enthusiastically starts up a new treatment program and after 1 or 2 years they lose enthusiasm or funding and the program stops. That’s a problem.”

##### Survey

Fifty-six percent of all health care providers and 25 % of all GPs indicated having sufficient knowledge to help/treat children with obesity. Only 11 % of GPs and 23 % of other health care providers were satisfied with the results of current obesity projects. Eighty-three percent of GPs and 85 % of all health care providers considered it important to receive feedback about treatment results from other professionals who treated these children and 68 % and 65 % respectively found it important that parents be reimbursed for treatment by their health insurance providers. More than 80 % of GPs and all health care providers indicated the need for a social map with an overview of lifestyle interventions to which obese children could be referred.

#### Component 4: continuity of care

##### Focus group/Interview

All health care providers indicated that there is currently no continuity of care for obese children. Also, structural funding is needed to provide continuity of care. Firstly, the target group needs time and attention, which cannot be given by the GPs due to lack of funding. As such, GPs cannot appoint extra human resources to tackle this problem. Secondly, there is a lack of long-term treatment programs to refer children to, as these programs are not covered by health insurance. The only option they have is to refer to a pediatrician who can provide prolonged care. But even the children that are referred to secondary care programs seem to lose motivation and drop out with no change in their long-term behavior. It is also unclear how many children that are referred actually visit the specialist.

Dietician: “Four hours of dietary advice annually. That’s actually reasonable, but then you can’t address the motivation process due to time constraints.”

Youth health care doctor: “The problem is that people do not always visit the specialist or just quit the treatment. You know, we don’t know how often, but quite regularly I think.”

##### Survey

Two-thirds of GPs and health care providers indicated a need for additional time to provide optimal treatment to obese children. Half of the GPs (55 %) and 62 % of all health care providers would require extra financial compensation to perform this task.

#### Component 5: multidisciplinary approach and case management

##### Focus groups/interview

All health care providers indicated that the current care of overweight and obese children is fragmented, lacks a collaborative component and does not resemble the multidisciplinary approach described in the integrated health care standard. More specifically, the pediatrician indicated that the GP is the missing link in current care. The health care providers indicated that a central care coordinator could be a good solution in terms of monitoring continuity of and cooperation in care. However, a central care coordinator is not available in the context of current care and appointing one would require structural funding. Although all health care providers recognized the importance of clear task rearrangement, they did not see any opportunities for change in the short term.

Youth health care doctor: “It is not the only task of the central care coordinator to motivate the patients, but to keep the other health care providers informed as well.”

Pediatrician: “The weak link in the current health care chain is the general practitioner. So basically, obese children without co-morbidities cannot be referred to primary care, unless you have found a motivated general practitioner.”

##### Survey

Twenty-seven percent of the health care providers agreed that they require clear task delegation between relevant health care providers. To provide care as described in the integrated health care standard, 75 % of GPs and 71 % of all health care providers found it important to receive feedback from the health care providers involved and a notification when a child is referred. According to more than half (64 %) of the health care providers, a central care coordinator is needed.

## Discussion

This study investigated the barriers to and needs for the implementation of the integrated health care standard on care of overweight and obese children as perceived by health care providers. We identified a number of important barriers to the implementation of the five key components of the integrated health care standard (i.e. reluctance to raise the subject of weight; lack of time for optimal treatment; lack of long-term interventions; no structured multidisciplinary approach; financial constraints and lack of feedback) and several needs (i.e. obesity knowledge and awareness; financial reimbursement; task rearrangement; feedback information and a central care coordinator). These barriers and needs are of great importance in defining strategies for the implementation of the integrated health care standard.

GPs experienced difficulties in identifying obese children and indicated a lack of competence in this area. They were reluctant to raise the issue of a child’s excessive weight due to several factors: fear of harming the relationship with the children and/or parents; expectations of non-compliance; no clear role identification; lack of knowledge; negative previous experience and insufficient obesity awareness. Similar barriers have been identified in other studies [16, 32–35]. It is important that GPs raise the subject because most parents do not seek support in dealing with overweight children [36]. Whereas GPs believe involvement in a patient’s weight management is part of their role, only a small group of GPs regularly provides care in this area, which may be due to their own (lack of) confidence and knowledge [34]. Previous research has indicated that parents of obese children are also reluctant to consult a GP due to a fear of being blamed for their child’s weight and a concern about their child’s mental well-being [36]. Parents want to protect their child from developing low self-esteem and some parents prefer that their child not be present when discussing his or her weight problem [37]. A systematic review of parental perception of overweight status in children found that more than half of parents are not able to identify their child as being overweight [38]. This is an important factor for health care providers as it indicates that they need to make parents more aware of obesity in their children. As is confirmed in the literature, training GPs in increased awareness and knowledge of obesity may result in improved identification and discussion of weight problems and diminishing the fear of harming the relationship [39].

All health care providers recognized the difficulties of treatment (i.e. lack of interventions, financial constrains); continuity of care (i.e. lack of monitoring) and a multidisciplinary approach (i.e. lack of coordination, absence of task rearrangements). Furthermore, our results show that health care providers are less motivated to provide treatment due to poor results from previous treatment and unmotivated children. The aforementioned difficulties related to the different components of the integrated health care standard were also reported in earlier studies [13–17, 39, 40]. Successful implementation of the integrated health care standard for the management of childhood obesity is in part dependent upon the identification of these barriers. As described in the literature, the critical problem is not the creation of an integrated health care standard, but the creation of support systems for its implementation at the level of primary health care [16, 40, 41]. The fact that these barriers have also been identified in previous research highlights the need to take them into consideration in the implementation process. The novel contribution of this study is the finding that the majority of health care providers see the appointment of a central care coordinator as a viable solution to these barriers.

This study provides qualitative and quantitative information on health care providers’ perceptions of the barriers to and needs for the implementation of integrated childhood obesity care. The open nature of the focus groups and the face-to-face, semi-structured interviews meant that the health care providers could offer more detailed viewpoints and raise issues that were of importance to them.

The internet survey provided more quantitative insight into how specific themes are important to health care providers and the differences between the views of the different health care providers. A limitation of our study is the low response rate of the internet survey. We previously speculated that the low response rate of the internet survey might have been due to our recruitment approach. We were concerned for a low response rate due to the fact that the health care workers were invited indirectly (not personally) to fill in the online questionnaire by health care organizations and services that tend to send many regular e-mails each week. Therefore, we decided to send a high number of invitations out in order to recruit a large enough sample. E-mail was chosen because it is cost effective, both in terms of time and money and the method yields fewer unanswered items than other modes [42]. We cannot speculate as to the way in which the low response rate may have influenced the results of the study. The internet survey had added value in that it showed that the health care providers’ perceived barriers and needs were less pronounced in the survey than in the interviews and the focus groups. However, a substantial part of the survey identified the same barriers as the qualitative part of the study did. This shows that qualitative research is useful in the identification of different points of view, but might overestimate the importance of these points of view. Another possible explanation is that the survey and interviews were subject to a selection bias. Another limitation of the study is that the health care providers were employed in a large urban area, which may limit the generalizability of our findings. However, in this area, health care providers have experience in the care of obese children because of the implementation of governmental programs and the increasing prevalence of childhood obesity. It is unlikely that barriers and needs differ in other regions.

## Conclusions

This study focused on context analysis as a first step in the implementation process of the integrated health care standard. We identified the central barriers and needs in each of the five key components of the integrated health care standard. The findings of this study suggest that the mere publication of the integrated health care standard is unlikely to elicit meaningful change in integrated childhood obesity care. In order to improve care, the next step is the development of strategies to change practice.

For progress to be made, emphasis should be placed on the awareness of health care providers of the barriers children and parents face during treatment. Strategies should be defined to elicit change at different levels (i.e. at the level of the individual care provider, the system and social setting) by using the five components of the integrated health care standard. Task rearrangements and coordination agreements also need to be made and feedback needs to be provided on the condition that financial reimbursement be available to improve integrated childhood obesity care.

## Additional files

Additional file 1: Key questions focus group. (DOCX 14 kb) Additional file 2: Topics interview guide. (DOCX 13 kb) Additional file 3: Overview of the survey results for all health care providers (GP responses separated). (DOCX 30 kb)

## Abbreviations

ANH-VUMC: Academic Network of General Practitioners of the VU University Medical Centre in Amsterdam
COREQ: consolidated criteria for reporting qualitative research
GG&GD: municipal youth health care organization
GP: general practitioner
GGR: weight-related health risk
Integrated health care standard: integrated health care standard for obesity
YHC: youth health care
