# Supplementary material for: Health care providers’ perceived barriers to and need for the implementation of a national integrated health care standard on childhood obesity in the Netherlands – a mixed methods approach
Source: BMC Health Serv Res. 2016 Mar 8;16:83. doi: 10.1186/s12913-016-1324-7 (PMC4784354; doi:10.1186/s12913-016-1324-7)
Supplement: Additional file 1: — Key questions focus group. (DOCX 14 kb) [file 12913_2016_1324_MOESM1_ESM.docx]

**Additional file 1: key questions focus group**

1. How important/urgent is it to treat children with obesity?
2. What are the barriers in current practice to identifying/treating children with obesity?
3. What are the factors in current practice that facilitate the identification/treatment of children with obesity?
4. What are the barriers to working with the integrated health care standard?
5. What factors facilitate working with the integrated health care standard?
6. What do you need to be able to work in accordance with the integrated health care standard?
