# Supplementary material for: Health care providers’ perceived barriers to and need for the implementation of a national integrated health care standard on childhood obesity in the Netherlands – a mixed methods approach
Source: BMC Health Serv Res. 2016 Mar 8;16:83. doi: 10.1186/s12913-016-1324-7 (PMC4784354; doi:10.1186/s12913-016-1324-7)
Supplement: Additional file 2: — Topics interview guide. (DOCX 13 kb) [file 12913_2016_1324_MOESM2_ESM.docx]

**Additional file 2: topics interview guide**

1. The current health care of children with obesity

1.1 Identification and diagnosis

1.2 Referral

1.3 Treatment

1.4 Cooperation🡪 Explain and introduce integrated care

1.5 Expertise

2. Guidelines

2.1 Central caregiver 🡪 that acts as a regular contact and has a coordinating role

2.2 Cooperation and integrated care agreements
2.3 individual care plan and central patient record

2.4 Possibilities for self-management

3. Comments/additions
