# Supplementary material for: Health care providers’ perceived barriers to and need for the implementation of a national integrated health care standard on childhood obesity in the Netherlands – a mixed methods approach
Source: BMC Health Serv Res. 2016 Mar 8;16:83. doi: 10.1186/s12913-016-1324-7 (PMC4784354; doi:10.1186/s12913-016-1324-7)
Supplement: Additional file 3: — Overview of the survey results for all health care providers (GP responses separated). (DOCX 30 kb) [file 12913_2016_1324_MOESM3_ESM.docx]

**Table 2: Overview of the survey results for all health care providers (GP responses separated).**

|  | **GPs**  **(N=57)** | | |  | | **All health care providers***  **(N=199)** | | | |
| --- | --- | --- | --- | --- | --- | --- | --- | --- | --- |
| **Question** | **Agree (%)** | **Disagree (%)** | **Neutral (%)** | **N/A (%)** | **Agree (%)** | | **Disagree (%)** | **Neutral (%)** | **N/A (%)** |
| **Interpretation of role in the care of obese children** |  |  |  |  |  | |  |  |  |
| I think it is important that children with obesity are treated. | 90 | 5 | 4 | 2 | 94 | | 2 | 2 | 1 |
| I see it as my role to treat children with obesity. | 40 | 9 | 49 | 2 | 54 | | 17 | 23 | 6 |
| I have experience in the care of obese children. | 37 | 30 | 32 | 2 | 58 | | 23 | 13 | 6 |
| **Component 1: identification** |  |  |  |  |  | |  |  |  |
| I am aware of the existence of the integrated health care standard. | 42 | 44 | 12 | 2 | 45 | | 40 | 13 | 2 |
| I am aware of the content of the integrated health care standard. | 26 | 54 | 18 | 2 | 35 | | 48 | 14 | 3 |
| I have a structured plan to help children with obesity. | 18 | 61 | 19 | 2 | 48 | | 32 | 15 | 5 |
| I have enough time to help children with obesity. | 11 | 60 | 26 | 4 | 43 | | 33 | 19 | 5 |
| I find it difficult to raise the topic of obesity. | 13 | 63 | 23 | 2 | 4 | | 85 | 12 |  |
| If your ‘agree’: Why do you find it difficult to discuss obesity? |  |  |  |  |  | |  |  |  |
| - Parents don’t request help with the child’s weight problem. | 75 |  |  |  | 100 | |  |  |  |
| - I worry that I will harm the relationship with child and/or parents. | 13 |  |  |  | 20 | |  |  |  |
| - I don’t want to harm the child/parents. | 50 |  |  |  | 60 | |  |  |  |
| - I worry about creating feelings of guilt in the child/parents. | 0 |  |  |  | 0 | |  |  |  |
| - I don’t have a lot to offer. | 38 |  |  |  | 20 | |  |  |  |
| - It takes up too much time. | 63 |  |  |  | 0 | |  |  |  |
| - Beliefs about obesity are culturally determined. | 38 |  |  |  | 0 | |  |  |  |
| Obese children do not come in for consultation. |  |  |  | 100 | 9 | |  |  | 91 |
| There is enough illness awareness among parents and children. | 8 | 73 | 17 | 2 | 6 | | 71 | 13 | 10 |
| Children are motivated to change their lifestyle. | 15 | 50 | 33 | 2 | 14 | | 36 | 38 | 12 |
| Parents and other family members are motivated to change their lifestyles. | 6 | 62 | 31 | 2 | 9 | | 49 | 30 | 12 |
| The socio-economic situation of the family plays a role in the onset and maintenance of obesity. | 89 | 6 | 4 | 2 | 79 | | 4 | 8 | 10 |
| **Component 2: diagnosis and risk stratification** |  |  |  |  |  | |  |  |  |
| I see it as my job to diagnose children with obesity. | 88 | 2 | 9 | 2 | 47 | | 19 | 20 | 15 |
| I track weight, height and BMI in patient records. | 46 | 38 | 16 |  | 69 | | 14 | 5 | 12 |
| I have sufficient tools with which to diagnose obesity. | 80 | 9 | 11 |  | 58 | | 13 | 14 | 15 |
| **Component 3: individual care plan and treatment** |  |  |  |  |  | |  |  |  |
| I treat obese children and their parents. | 30 | 70 |  |  | 51 | | 49 |  |  |
| I refer children with obesity to local interventions. | 60 | 40 |  |  | 50 | | 50 |  |  |
| I have enough knowledge to help children with obesity. | 25 | 25 | 49 | 2 | 56 | | 16 | 23 | 5 |
| I am satisfied with the results of interventions/ treatment. | 11 | 36 | 35 | 18 | 23 | | 16 | 30 | 31 |
| I am aware of current interventions for children with obesity. | 51 | 26 | 24 |  | 58 | | 24 | 12 | 7 |
| I have a good social map, so I know where to refer children. | 46 | 16 | 38 |  | 59 | | 18 | 15 | 8 |
| The costs of the intervention/ treatment are acceptable to parents. | 11 | 13 | 51 | 26 | 21 | | 11 | 33 | 35 |
| The health care providers with whom I work provide me with sufficient feedback information. | 23 | 37 | 37 | 4 | 37 | | 18 | 28 | 16 |
| **Component 4: continuity of care** |  |  |  |  |  | |  |  |  |
| I am satisfied with the duration of the interventions/treatment. | 15 | 29 | 42 | 15 | 28 | | 13 | 32 | 28 |
| The waiting list for intervention/treatment is acceptable. | 24 | 20 | 38 | 18 | 37 | | 8 | 22 | 33 |
| The intervention/treatment has enough follow-up | 11 | 16 | 47 | 26 | 29 | | 11 | 28 | 33 |
| Parents have sufficient pedagogical skills related to maintaining a healthy weight in their children. | 4 | 81 | 12 | 4 | 3 | | 67 | 19 | 2 |
| **Component 5: multidisciplinary approach** |  |  |  |  |  | |  |  |  |
| I work closely with other health care providers. | 37 | 25 | 39 |  | 53 | | 22 | 14 | 12 |
| The task delegation between health care providers is clear to me. | 19 | 35 | 42 | 4 | 54 | | 16 | 15 | 16 |
| I give feedback information to the health care providers with whom I work. | 27 | 27 | 42 | 4 | 60 | | 7 | 17 | 16 |
| There is enough contact between health care providers involved. | 8 | 52 | 35 | 6 | 29 | | 28 | 23 | 19 |
| There are too many health care providers involved in the care of children with obesity. | 15 | 46 | 31 | 8 | 16 | | 35 | 29 | 20 |
| I find It useful that there is an integrated health care standard for obesity. | 74 | 2 | 22 | 2 | 87 | | 1 | 11 | 2 |
| I agree with the integrated health care standard that care for children with obesity should be demand-driven instead of supply driven. | 56 | 8 | 32 | 4 | 67 | | 5 | 26 | 3 |
| I think it is good that there is an emphasis on a family approach during intervention. | 98 |  | 2 |  | 96 | | 1 | 1 | 2 |
| I think it is good that there is a multidisciplinary approach. | 96 | 2 | 2 |  | 94 | | 2 | 2 | 2 |
| I think it is good that a central care coordinator is appointed. | 82 | 2 | 14 | 2 | 90 | | 2 | 6 | 3 |
| It is problematic that the integrated health care standard provides no description of who should perform the role of the central caregiver. | 26 | 18 | 54 | 2 | 42 | | 11 | 42 | 5 |
| It is problematic that there is no clear division of labor between relevant health care providers. | 22 | 16 | 58 | 4 | 27 | | 11 | 58 | 4 |
| I like the fact that data is stored in a central patient record. | 46 | 18 | 34 | 2 | 66 | | 7 | 23 | 5 |
| I like the fact that the individual health care plan is tailored to the wishes and needs of children and parents. | 82 | 2 | 12 | 4 | 82 | | 4 | 10 | 5 |

| **As a health care provider, what do you need for optimal treatment of children with obesity?** | **GPs**  **(N=71)** | | | | | | | | | **All health care providers***  **(N=98)** | | | | | | | | | | | | | | |
| --- | --- | --- | --- | --- | --- | --- | --- | --- | --- | --- | --- | --- | --- | --- | --- | --- | --- | --- | --- | --- | --- | --- | --- | --- |
|  | **Important (%)** | **Not important(%)** | | **Neutral (%)** | **N/A (%)** | | **Important (%)** | | | | **Not important (%)** | | | | | **Neutral (%)** | | | | **N/A (%)** | | |  |  |
| **Component 1: identification** |  |  |  | | |  | | | |  | | | |  | | | |  | | | |  | | |
| More awareness of the health risks of obesity in health care. | 34 | 27 | 37 | | | 3 | | 50 | | | | 14 | | | | | 32 | | | | 4 | | |  |
| More knowledge. | 47 | 18 | 32 | | | 3 | | 61 | | | | 11 | | | | | 25 | | | | 3 | | |  |
| Improved identification by general practitioners. | 56 | 10 | 31 | | | 3 | | 66 | | | | 8 | | | | | 22 | | | | 3 | | |  |
| Improved identification by YHC-doctors. | 62 | 11 | 24 | | | 3 | | 64 | | | | 8 | | | | | 21 | | | | 6 | | |  |
| Information leaflets for parents and children. | 75 | 10 | 11 | | | 4 | | 70 | | | | 5 | | | | | 21 | | | | 3 | | |  |
| Availability of interpreters. | 44 | 10 | 44 | | | 3 | | 62 | | | | 6 | | | | | 29 | | | | 3 | | |  |
| An annual screening of weight for children aged 4-12 years. | 63 | 10 | 23 | | | 4 | | 69 | | | | 3 | | | | | 25 | | | | 3 | | |  |
| More political influence to promote a healthy lifestyle. | 69 | 13 | 16 | | | 3 | | 66 | | | | 12 | | | | | 18 | | | | 3 | | |  |
| More education at school about healthy food. | 83 | 10 | 6 | | | 2 | | 85 | | | | 4 | | | | | 8 | | | | 3 | | |  |
| **Component 2: diagnosis and risk stratification** |  |  |  | | |  | | |  | | | |  | |  | | | |  | | | |  |  |
| Diagnostic tools for the measurement of weight, height and BMI. | 35 | 27 | 35 | | | 3 | | | 49 | | | | 15 | | | | 31 | | | | 5 | | |  |
| Diagnostic tools to identify psychological problems. | 66 | 7 | 24 | | | 3 | | | 78 | | | | 5 | | | | 14 | | | | 3 | | |  |
| **Component 3: individual care plan and treatment** |  |  |  | | |  | | |  | | | |  | | | |  | | | |  | | |  |
| Feedback from health care providers involved. | 83 | 7 | 7 | | | 3 | | | 85 | | | | 5 | | | | 7 | | | | 3 | | |  |
| A social map that is makes it clear to whom I can refer an obese child. | 83 | 9 | 6 | | | 3 | | | 81 | | | | 6 | | | | 10 | | | | 3 | | |  |
| Knowledge/feedback about results of treatment/projects. | 76 | 14 | 7 | | | 3 | | | 82 | | | | 6 | | | | 7 | | | | 5 | | |  |
| Parents should be totally reimbursed for treatment by the health insurance provider. | 68 | 11 | 18 | | | 3 | | | 65 | | | | 8 | | | | 24 | | | | 3 | | |  |
| Availability of child psychologists. | 59 | 6 | 31 | | | 4 | | | 62 | | | | 8 | | | | 26 | | | | 4 | | |  |
| **Component 4: continuity of care** |  |  |  | | |  | | |  | | | |  | | | |  | | | |  | | |  |
| More time. | 65 | 14 | 11 | | | 10 | | | 66 | | | | 7 | | | | 22 | | | | 4 | | |  |
| Extra financial compensation for health care providers involved. | 55 | 11 | 31 | | | 3 | | | 62 | | | | 6 | | | | 29 | | | | 3 | | |  |
| **Component 5: multidisciplinary approach** |  |  |  | | |  | | |  | | | |  | | | |  | | | |  | | |  |
| Recruitment of a central care coordinator. | 56 | 31 | 10 | | | 3 | | | 64 | | | | 5 | | | | 22 | | | | 3 | | |  |
| A notification when a health care provider refers a child to me, so I am aware of the referral. | 75 | 6 | 17 | | | 3 | | | 71 | | | | 11 | | | | 13 | | | | 4 | | |  |
| Establishment of obesity clinics for parents with obese children. | 28 | 27 | 42 | | | 3 | | | 51 | | | | 18 | | | | 28 | | | | 3 | | |  |

**N/A= not applicable**

* Health care providers included are YHC workers, pediatricians, dieticians, psychologists, physiotherapists and others (e.g. obesity coordinator).
